# Apoptosis in a Whitefly Vector Activated by a Begomovirus Enhances Viral Transmission

**DOI:** 10.1128/mSystems.00433-20

**Published:** 2020-09-22

**Authors:** Xin-Ru Wang, Chao Wang, Fei-Xue Ban, Murad Ghanim, Li-Long Pan, Li-Xin Qian, Yin-Quan Liu, Xiao-Wei Wang, Shu-Sheng Liu

**Affiliations:** a Ministry of Agriculture Key Laboratory of Molecular Biology of Crop Pathogens and Insects, Institute of Insect Sciences, Zhejiang University, Hangzhou, China; b Department of Entomology, Agricultural Research Organization, The Volcani Center, Bet Dagan, Israel; University of California, Davis

**Keywords:** apoptosis, DNA virus, begomovirus, insect vector, transmission, whitefly

## Abstract

Apoptosis is generally considered the first line of defense against viral infection. However, the role of apoptosis in the interactions between plant viruses and their insect vectors has rarely been investigated. By studying plant DNA viruses of the genus *Begomovirus* within the family *Geminiviridae*, which are transmitted by whiteflies of the Bemisia tabaci species complex in a persistent manner, we revealed that virus-induced apoptosis in insect vectors can facilitate viral accumulation and transmission. We found that infection with tomato yellow leaf curl virus activated the apoptosis pathway in *B. tabaci*. Suppressing apoptosis by inhibitors or silencing *caspase-3* significantly reduced viral accumulation, while the activation of apoptosis increased viral accumulation *in vivo*. Moreover, the positive effect of whitefly apoptosis on virus accumulation and transmission was not due to its cross talk with the autophagy pathway that suppresses begomovirus infection in whiteflies. We further showed that viral replication, rather than the viral coat protein, is likely the critical factor in the activation of apoptosis by the virus. These novel findings indicate that similarly to many animal and a few plant RNA viruses, plant DNA viruses may activate apoptosis in their insect vectors leading to enhanced viral accumulation and transmission.

**IMPORTANCE** Of the approximately 1,100 known plant viruses, about one-third are DNA viruses that are vectored by insects. Plant virus infections often induce cellular and molecular responses in their insect vectors, which can, in many cases, affect the spread of viruses. However, the mechanisms underlying vector responses that affect virus accumulation and transmission are poorly understood. Here, we examined the role of virus-induced apoptosis in the transmission of begomoviruses, a group of single-stranded plant DNA viruses that are transmitted by whiteflies and cause extensive damage to many crops worldwide. We demonstrated that virus infection can induce apoptosis in the insect vector conferring protection to the virions from degradation, leading to enhanced viral accumulation and transmission to host plants. Our findings provide valuable clues for designing new strategies to block the transmission of insect-vectored plant viruses, particularly plant DNA viruses.

## Abstract (precis)

Of the approximately 1,100 known plant viruses, about one-third are DNA viruses that are vectored by insects. Plant virus infections often induce cellular and molecular responses in their insect vectors, which can, in many cases, affect the spread of viruses. However, the mechanisms underlying vector responses that affect virus accumulation and transmission are poorly understood. Here, we examined the role of virus-induced apoptosis in the transmission of begomoviruses, a group of single-stranded plant DNA viruses that are transmitted by whiteflies and cause extensive damage to many crops worldwide. We demonstrated that virus infection can induce apoptosis in the insect vector conferring protection to the virions from degradation, leading to enhanced viral accumulation and transmission to host plants. Our findings provide valuable clues for designing new strategies to block the transmission of insect-vectored plant viruses, particularly plant DNA viruses.

## INTRODUCTION

Of the approximately 1,100 known plant viruses, about one-third are DNA viruses that are transmitted exclusively by insects, particularly whiteflies (1). DNA viruses of the genus *Begomovirus*, family *Geminiviridae*, infect hundreds of plant species, including many important crops, and cause devastating damage to agricultural production in tropical and subtropical regions (2, 3). Begomoviruses are exclusively transmitted by whiteflies of the Bemisia tabaci species complex in a persistent manner (4). The virions, which are ingested by whiteflies along with phloem sap of infected plants through the stylet, move to the midgut, and then come across the midgut wall to the hemocoel, and finally to the salivary glands where they are finally excreted to plants with the saliva during feeding (5). Several begomoviruses have been shown to induce a variety of cellular, molecular, and behavioral responses in their insect vectors, which may affect the survival of the vector and the spread of the virus (6). However, the mechanisms underlying these responses in the vectors are poorly understood.

Begomoviruses have evolved complex relationships with their insect vectors (7). Previous reports indicate that some begomoviruses, such as tomato yellow leaf curl virus (TYLCV) and tomato yellow leaf curl China virus (TYLCCNV), may exert adverse effects on their whitefly vectors and thus show some intriguing resemblances to entomopathogenic viruses (8, 9). Begomoviruses, once acquired by whiteflies, are present in their whitefly vectors for most, if not all, of the insects’ life (5). Furthermore, TYLCV, a devastating begomovirus that has caused heavy losses to tomato crops worldwide, has been reported to replicate in its insect vectors. By analyzing the expression of viral *V1* and *C3* genes in both orientations of virus DNA, Pakkianathan et al. obtained evidence of TYLCV replication in whiteflies (10). In addition, circumstantial evidence of TYLCV replication is provided by the successful transovarial transmission of the virus from one generation to the next of the whitefly vector (11–14). Equally important, case studies also showed that both replication and transovarial transmission of TYLCV in whiteflies may be conditional and intermittent (13, 14), and the replication of TYLCV occurs mainly in the salivary glands of the whitefly vector (15).

Apoptosis is a highly regulated and well-studied defense response involving blebbing, cell shrinkage, nuclear fragmentation, chromosomal DNA fragmentation, and global mRNA decay (16). An evolutionarily conserved feature of apoptosis is the activation of caspases (17). As one of the most notable players in the apoptotic scene, the cytochrome *c* release from mitochondrion into the cytosol could initiate the activation cascade of caspases (18). Apoptosis plays a vital role in animal responses to virus infection (19), and its role in viral replication and spread varies depending on the virus, the duration of infection, the cell type, and other factors. In some cases, the induction of apoptotic cell death counteracts deployment of host cellular components by the virus for propagation (20, 21), while in others, apoptosis induced by viral infections preserves/promotes viral infection and replication (22–25). However, the role of apoptosis in the interactions between insect vectors and plant viruses is yet poorly known. For example, do persistently transmitted viruses trigger apoptosis in their insect vectors, and if so, what are the apoptotic molecular responses in the insects to virus infection, and what are the effects of apoptosis on the fate of viral particles in an insect vector?

In addition to apoptosis, viruses are capable of inducing autophagy, an evolutionarily conserved vacuolar, self-eating mechanism in which cellular components are recycled or degraded in the lysosomal compartment (26). In a previous study, we observed autophagy in whiteflies induced by TYLCV infection and autophagy-associated reduction of viral accumulation (12). Here, we show that TYLCV infection can trigger apoptosis in its insect vector, and this activation facilitates viral accumulation and transmission. We also demonstrate that no cross talk occurs between autophagy and apoptosis pathways after TYLCV infection. These results provide novel insights into insect responses to the infection of plant viruses and their effects on virus transmission.

## RESULTS

### Apoptosis is activated in whiteflies in response to TYLCV infection.

**FIG 1:**
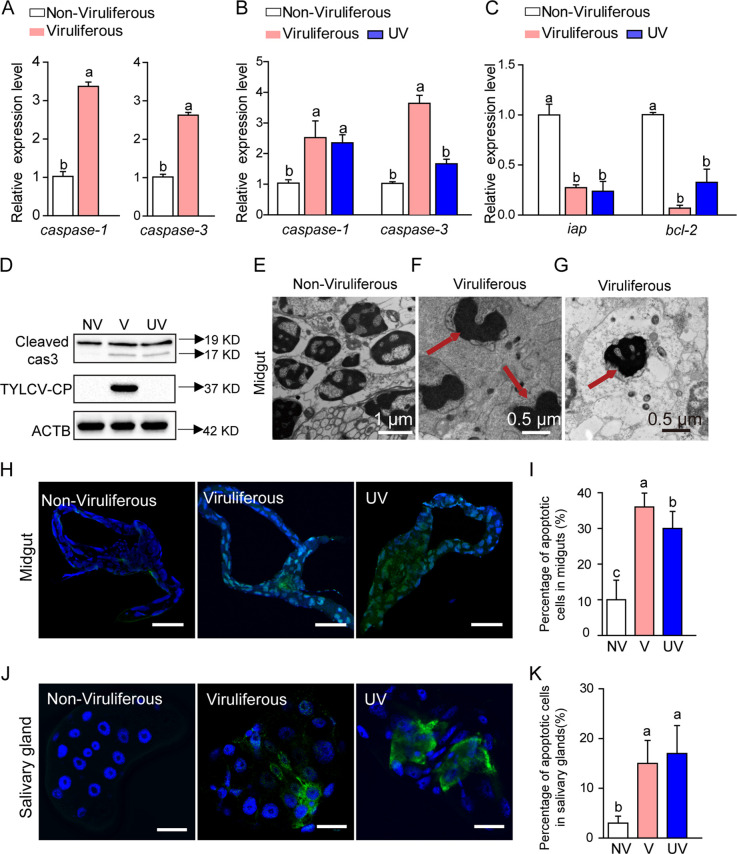
TYLCV infection activates apoptosis in whiteflies. Expression of caspase genes in nonviruliferous and viruliferous whiteflies. (A) Relative expression levels of *caspase-1* and *caspase-3* were measured by qRT-PCR with *ACTB* and *EF-1a* expression as the internal control. (B and C) Expression of apoptosis-related genes in nonviruliferous, viruliferous, and UV-treated whiteflies. Relative expression levels of *caspase-1*, *caspase-3*, *iap*, and *bcl-2* were measured by qRT-PCR with *ACTB* and *EF-1a* expression as the internal control. (D) Immunoblot analysis of cleaved-caspase-3 and viral coat protein (CP) in nonviruliferous (NV), viruliferous (V), and UV-treated (UV) whiteflies. ACTB is the loading control in Western blots. (E, F, and G) Images of nuclei of midgut cells of nonviruliferous (E) and viruliferous (F and G) whiteflies observed under a transmission electron microscope; 200 midguts were dissected and observed for nonviruliferous and viruliferous whiteflies, respectively, and representative images are shown. Arrows in panel F indicate coiled chromatin in the nucleus, and the arrow in panel G indicates cavitations in the nucleus. (H and J) Images showing apoptosis in the nuclei of midgut cells (H) and salivary gland cells (J) of nonviruliferous, viruliferous, and UV-treated (UV) whiteflies; guts were dissected, fixed, and labeled with TUNEL to show apoptosis (green), and blue DAPI staining shows the nuclei. Bar, 100 μm. For each of the three treatments, 10 midguts and salivary glands were dissected, and representative images are shown. (I and K) Proportions of apoptotic cells (number of TUNEL-positive cells/number of DAPI-positive cells) in midguts (I) and salivary glands (K). In panels A, B, C, I, and K, data are mean ± SE, and different letters above the columns in each diagram indicate significant differences (ANOVA, followed by Bonferroni test).

To examine whether apoptosis is triggered upon TYLCV infection, we placed whiteflies on TYLCV-infected and uninfected tomato plants to feed for 24 h and then examined the expression of caspase genes in viruliferous and nonviruliferous whiteflies. The expression of two caspase genes (*caspase-1* and *caspase-3*) increased significantly in TYLCV-infected whiteflies (Fig. 1A). To examine whether the increase of caspase gene expression was caused indirectly by the physiological change of host plant following TYLCV infection rather than the virus *per se*, we placed whiteflies to feed on virus-infected tomato plants for 24 h and then transferred the insects to feed on cotton, a nonhost plant of TYLCV, for 120 h. Compared to the control, nonviruliferous whiteflies, the treated whiteflies showed significantly higher expression of *caspase-1* and *caspase-3* but lower expression of some other genes, such as *bcl-2* encoding Bcl-2 protein that inhibits apoptosis via interactions with the proapoptotic proteins, and *iap* encoding inhibitors of apoptosis that can block the functioning of caspases (Fig. 1B and C). Cell apoptosis can also be caused by DNA damage inflicted by UV radiation (27). As a positive control, whiteflies exposed to UV radiation for 6 h showed higher expression of *caspase-1* but lower expression of *iap* and *bcl-2* (Fig. 1B and C). Further analysis using Western blotting (28) showed an apparent increase in the level of cleaved-caspase-3, an apoptotic cell-death-specific protein marker, in viruliferous whiteflies but not in nonviruliferous whiteflies (Fig. 1D). Previous studies have demonstrated that cotton is a highly suitable host plant for whiteflies, and the transfer from tomato to cotton does not generate stress to the insects (12, 29). These results suggest that virus infection directly activated whitefly apoptosis.

To further analyze the induction of apoptosis in the vector by viral infection, we examined the morphology of whitefly midgut cells with or without TYLCV infection. Under electron microscopy, we found abundant apoptotic cells in the midguts of virus-infected whiteflies. The chromatin in the nucleus was heavily coiled (Fig. 1F), and many cavitations were found in the nucleus of virus-infected midguts (Fig. 1G). We also detected DNA fragmentation in whitefly midguts and salivary glands by terminal deoxynucleotidyl transferase (TdT)-mediated dUTP nick-end labeling (TUNEL) (30, 31). More apoptotic cells were detected in midguts of both viruliferous and UV-treated whiteflies than in nonviruliferous whiteflies (Fig. 1H and I). Colocalization experiments with TUNEL and TYLCV coat protein (CP) on the same sample of salivary glands showed that apoptosis was activated where the virus was present (Fig. 1J and K). Similar colocalization of TUNEL with TYLCV CP was observed in whitefly midgut (see Fig. S1 in the supplemental material). These observations indicate that the activation of apoptosis is strongly correlated with the presence of the virus. To investigate whether mitochondria were involved in TYLCV-induced apoptosis, we examined cytochrome *c* release by Western blotting. Cytochrome *c* accumulation in the cytosol was higher in viruliferous than in nonviruliferous whiteflies (Fig. S2). Altogether, these results strongly suggest that TYLCV infection activates the whitefly apoptosis pathway.

10.1128/mSystems.00433-20.1 FIG S1 Colocalization for TUNEL and CP in the gut of whiteflies. Localization of TUNEL and CP on the same sample. Bar, 100 μm. Blue indicates DAPI staining of the nuclei. For each treatment, 20 midguts were dissected and similar trends were observed. Download FIG S1, TIF file, 1.2 MB. Copyright © 2020 Wang et al. 2020 Wang et al. This content is distributed under the terms of the Creative Commons Attribution 4.0 International license.

10.1128/mSystems.00433-20.2 FIG S2 Cytochrome *c* is released in whiteflies after TYLCV infection. Immunoblot analysis of cytochrome *c* in the cytosol of whiteflies that fed on TYLCV-infected tomato plants for 24 h and then transferred to feed on cotton plants for 120 h. ACTB is the loading control in the Western blots. Download FIG S2, TIF file, 0.4 MB. Copyright © 2020 Wang et al. 2020 Wang et al. This content is distributed under the terms of the Creative Commons Attribution 4.0 International license.

### Inhibiting or inducing apoptosis affects TYLCV accumulation in whiteflies.

**FIG 2:**
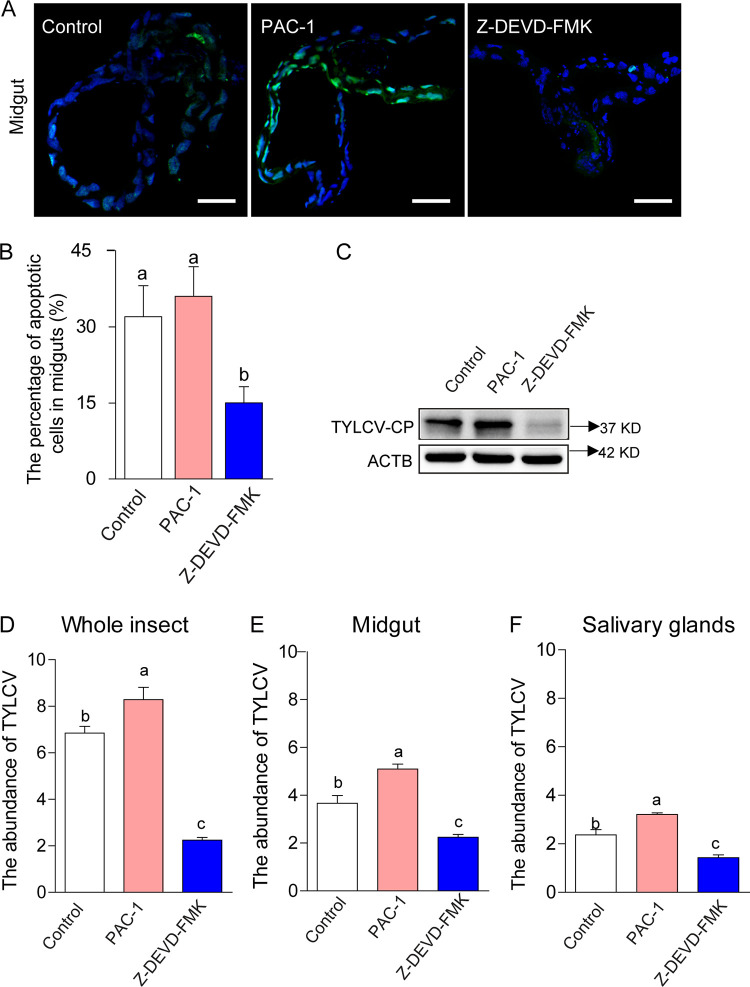
Effects of apoptosis on TYLCV accumulation in whiteflies. After feeding on TYLCV-infected tomato plants for 24 h, whiteflies were treated with apoptosis inhibitor Z-DEVD-FMK or apoptosis activator PAC-1 or with DMSO control for 12 h and then transferred to feed on cotton plants for 120 h before being collected for analysis. (A) Images of guts of whiteflies that were dissected, fixed, and labeled with TUNEL to show apoptosis (green). For each of the three treatments, 10 midguts were dissected, and representative images are shown. Blue DAPI staining corresponds to the nuclei. Bar, 100 μm. (B) Proportions of apoptotic cells (number of TUNEL-positive cells/number of DAPI-positive cells) in each midgut; each of the three treatments had three replicates with 10 whiteflies in each replicate. (C) Immunoblot analysis of TYLCV CP in whiteflies. ACTB is the loading control in the Western blots. (D, E, and F) The abundance of TYLCV genomic DNA as measured by qPCR in the whitefly whole body (D), midguts (E), and salivary glands (G); 20 whiteflies were measured in each of the 3 treatments of panels D, E, and F. In panels B, D, E, and F, data are mean ± SE, and different letters above the columns in each diagram indicate significant differences (ANOVA, followed by Bonferroni test).

We then examined the role of apoptosis in TYLCV interactions with the whitefly vector. After feeding on the virus-infected tomato plants for 24 h, the whiteflies were fed with the specific caspase-3 inhibitor, Z-DEVD-FMK (32), or an activator of procaspase-3, PAC-1 (33). Using TUNEL assay, we detected a significant reduction of apoptotic cells in the midguts of Z-DEVD-FMK-treated whiteflies and a slight increase of apoptotic cells in the midguts of PAC-1-treated whiteflies, compared to the control (Fig. 2A and B). TYLCV CPs decreased with the presence of the apoptosis inhibitor but increased with the presence of the apoptosis activator (Fig. 2C). Quantitative real-time PCR (qPCR) results showed, compared with the control, a significant increase in the accumulation of TYLCV genomic DNA in PAC-1-treated whiteflies but a significant decrease in Z-DEVD-FMK-treated whiteflies, when the whole body, midgut, and salivary glands were examined, respectively (Fig. 2D to F).

**FIG 3:**
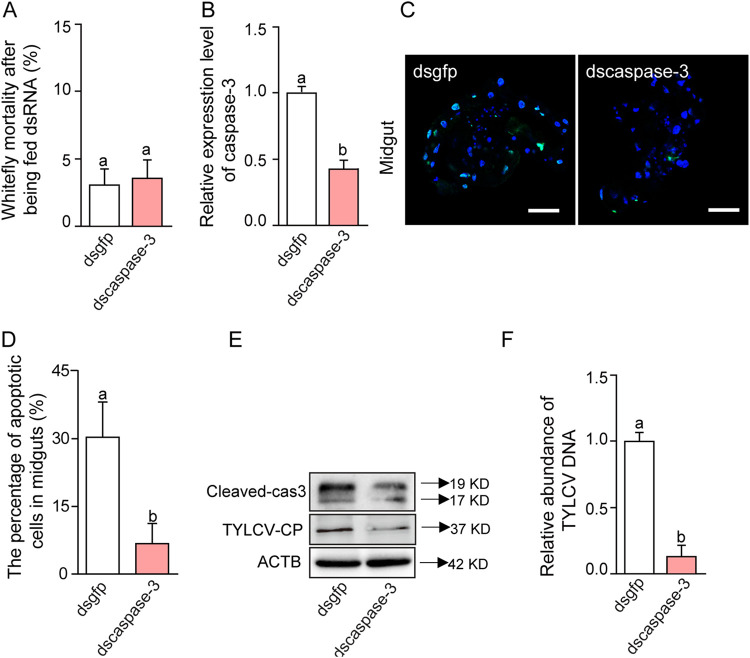
Silencing *caspase-3* inhibits apoptosis in whiteflies. Effects of silencing whitefly *caspase-3* gene on TYLCV (treatment with ds*caspase-3* versus ds*gfp* as control). (A) Mortality: percentage of dead adults after feeding on dsRNA solutions for 2 days. (B) Efficiency of RNAi. (C) Images of guts of viruliferous whiteflies that were treated with ds*caspase-3* or ds*gfp* and then were dissected, fixed, and labeled with TUNEL to show apoptosis (green). Blue DAPI staining shows the nuclei. For each treatment, 10 midguts were dissected, and representative images are shown. Bar, 100 μm. (D) Proportions of apoptotic cells (number of TUNEL-positive cells/number of DAPI-positive cells) in midguts. (E) Immunoblot analysis of TYLCV CP and cleaved-caspase-3 in whiteflies. (F) TYLCV genomic DNA as measured by qPCR. In panels A, B, D, and F, data are mean ± SE, and different letters above the columns in each diagram indicate significant differences (Student’s two-tailed *t* test).

Next, we utilized RNA-mediated interference (RNAi) to verify our findings further, and the effector caspase-3 was taken as the candidate gene for silencing. After the whiteflies had fed on double-stranded RNA (dsRNA) for 2 days, their survival was not affected, while the expression of *caspase-3* was reduced remarkably (Fig. 3A and B). When the whiteflies had fed on the virus-infected tomato plants for 24 h, the proportion of apoptotic cells in the midgut of the *caspase-3* gene-silenced whiteflies was significantly reduced (Fig. 3C and D), and the accumulation of viral CP was also substantially reduced compared with the control group that was fed with the double-strand RNA against green fluorescent protein (dsgfp) (Fig. 3E). Consistent with these observations, silencing *caspase-3* strongly reduced the quantity of virus genomic DNA in whiteflies (Fig. 3F). Our data suggested that inhibition of apoptosis could lead to a reduction of viral DNA and CP in whiteflies. Taken together, these observations show that virus-induced apoptosis enhances viral accumulation in whiteflies.

### Apoptosis affects the retention and transmission of TYLCV by whiteflies.

**FIG 4:**
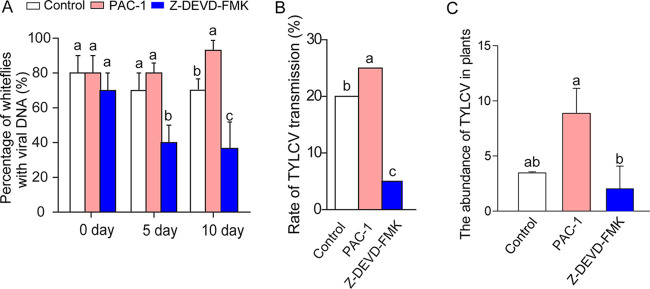
Effects of apoptosis on TYLCV transmission. Whiteflies were fed on virus-infected tomato plants for 24 h to acquire the virus, treated with apoptosis inhibitor Z-DEVD-FMK, with apoptosis activator PAC-1, or with DMSO as a control for 12 h, and then either transferred to feed on cotton plants for various durations before being collected for viral DNA analysis (A) or transferred singly to feed for 48 h on tomato plants for TYLCV transmission (B and C). (A) Retention of virus (% whiteflies with viral DNA, detected by PCR) over different time intervals. (B) Percentage of tomato plants showing typical symptoms of TYLCV infection; different letters above the columns indicate significant differences (Mann-Whitney U-tests). (C) Relative abundance of viruses in tomato plants, as measured by qPCR and normalized with tomato actin. In panels A and C, data are mean ± SE, and different letters above the columns in each diagram indicate significant differences (ANOVA, followed by Bonferroni test).

To investigate the role of apoptosis in the transmission of TYLCV by whiteflies, we fed whiteflies on virus-infected tomato plants and then immediately treated them with either Z-DEVD-FMK or PAC-1. We monitored the viral retention in whiteflies after they had fed on either of the two chemicals. TYLCV infection was not markedly affected by suppressing or enhancing apoptosis at 0 day (Fig. 4A). However, after transferring viruliferous whiteflies to feed on cotton plants for 5 days and 10 days viral DNA was detected in 40% and 35% of the whiteflies treated with the apoptosis inhibitor Z-DEVD-FMK, but in 80% and 92% of the whiteflies treated with the apoptosis activator PAC-1, respectively. Compared with the control, the increase of the percentage of viruliferous whiteflies associated with apoptosis activation was significant on day 10, and the reduction of the percentage of whiteflies associated with apoptosis inhibition was significant on both day 5 and day 10 (Fig. 4A). Furthermore, activating apoptosis increased, but inhibiting apoptosis reduced, the rate of virus transmission (Fig. 4B). The average abundance of TYLCV was significantly higher in tomato plants fed by activator-treated whiteflies than that in the plants fed by inhibitor-treated whiteflies (Fig. 4C). These results suggest that the activation of apoptosis facilitates, while inhibition of apoptosis suppresses, viral transmission by whiteflies.

### CP is not the trigger of apoptosis activation.

To investigate the key viral factor(s) that activates whitefly apoptosis, we infected whiteflies with another two begomoviruses: tomato yellow leaf curl China virus (TYLCCNV), which replicates in whiteflies after a short-interval virus acquisition by the insects on virus-infected tomato, and papaya leaf curl China virus (PalCuCNV), which does not replicate in whiteflies (12). The level of cleaved-caspase-3 in TYLCCNV- and PalCuCNV-fed whiteflies was monitored using Western blotting. Following TYLCCNV infection, there was an obvious accumulation of cleaved-caspase-3 (Fig. S3A), which was not found after PalCuCNV infection (Fig. S3B). Meanwhile, we also infected whiteflies with PalCuCNV and then treated them with PAC-1. More apoptotic cells and higher accumulation of PalCuCNV CP and genomic DNA were observed in the midguts of whiteflies treated with PAC-1, compared with the control (Fig. S4). As PalCuCNV cannot replicate in whitefly vectors, the increase of PalCuCNV after apoptosis activation suggests that apoptosis could help viral particles escape from degradation.

10.1128/mSystems.00433-20.3 FIG S3 TYLCCNV-induced apoptosis in whitefly. (A) Detection of TYLCCNV CP and cleaved-caspase-3 using Western blot analysis in nonviruliferous whiteflies and whiteflies that fed on TYLCCNV-infected tomato plants for 24 h and then transferred to feed on cotton plants for 120 h. ACTB is the control for loading. (B) Detection of PalCuCNV CP and cleaved-caspase-3 using Western blot analysis in nonviruliferous whiteflies and whiteflies that fed on PalCuCNV-infected tomato for 24 h and then transferred to feed on cotton plants for 120 h. ACTB is the control for loading. Download FIG S3, TIF file, 0.8 MB. Copyright © 2020 Wang et al. 2020 Wang et al. This content is distributed under the terms of the Creative Commons Attribution 4.0 International license.

10.1128/mSystems.00433-20.4 FIG S4 Virus increase following induction of apoptosis in PalCuCNV-infection whiteflies. Whiteflies were placed to feed on PalCuCNV-infected tomato plants for 24 h for virus acquisition, treated with apoptosis inducer PAC-1 or untreated using artificial diet for 12 h, and then transferred to feed on cotton plants for 120 h before being collected for analysis. (A) Images of guts of whiteflies that were treated with the apoptosis inducer PAC-1 or untreated, dissected, fixed, and labeled with TUNEL to show apoptosis. For treated and untreated whiteflies, 10 midguts were dissected, respectively, and representative images are shown. Bar, 100 μm. (B) Proportions of apoptotic cells (number of TUNEL-positive cells/number of DAPI-positive cells) in midguts. (C) Immunoblot analysis of PalCuCNV CP in whiteflies treated with PAC-1 or untreated. ACTB is the control for loading in Western blots. (D) PalCuCNV genomic DNA measured by qPCR in nonviruliferous whiteflies and whiteflies treated with PAC-1. In panels B and D, data are mean ± SD, and different letters above the two columns in each of the diagrams indicate significant difference (Student’s two-tailed *t* test). Download FIG S4, TIF file, 0.8 MB. Copyright © 2020 Wang et al. 2020 Wang et al. This content is distributed under the terms of the Creative Commons Attribution 4.0 International license.

**FIG 5:**
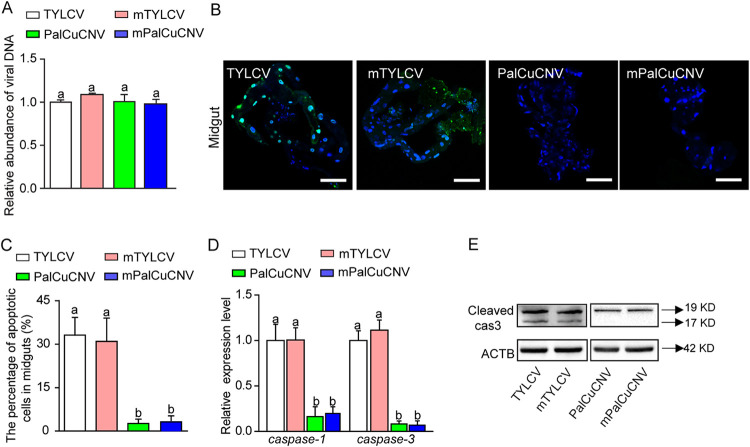
CP is not essential for activating apoptosis. Whiteflies were fed on wild-type TYLCV (TYLCV)-, mutant TYLCV (mTYLCV)-, wild-type PalCuCNV (PalCuCNV)-, or mutant PalCuCNV (mPalCuCNV)-infected tomato plants for 24 h and then collected for virus genomic DNA analysis (A) or transferred to feed on cotton plants for 120 h to be collected for apoptosis analysis (B to E). (A) Relative abundance of virus genomic DNA as measured by qPCR. (B) Images of guts of whiteflies in the four treatments that were dissected, fixed, and labeled with TUNEL to show apoptosis (green). Blue DAPI staining shows the nuclei. For each of the four treatments, 10 midguts were dissected, and representative images are shown. Bar, 100 μm. (C and D) Proportions of apoptotic cells (number of TUNEL-positive cells/number of DAPI-positive cells) in midguts (C) and relative expression levels of *caspase-1* and *caspase-3* as measured by qRT-PCR with *ACTB* and *EF-1a* expression as the internal control in the four treatments (D). (E) Immunoblot analysis of cleaved-caspase-3 in whiteflies. ACTB is the loading control in Western blots. In panels A, C, and D, data are mean ± SE, and different letters above the columns in each diagram indicate significant differences (ANOVA, followed by Bonferroni test).

To examine whether the viral CP was related to the activation of apoptosis, we exchanged a partial CP sequence of TYLCV with that of PalCuCNV and examined the staining of apoptotic cells after infection with the two CP mutant viruses (34). When the whiteflies had fed on wild-type TYLCV-infected and mutant TYLCV (mTYLCV)-infected tomato plants, the quantities of TYLCV they acquired were similar; the quantities of TYLCV acquired by the whiteflies feeding on wild-type PalCuCNV-infected and mutant PalCuCNV (mPalCuCNV)-infected tomato plants were likewise similar (Fig. 5A). Notably, apoptosis was strongly induced in whiteflies exposed to both wild-type TYLCV and mTYLCV, whereas few apoptotic cells were found in whiteflies infected with PalCuCNV and mPalCuCNV (Fig. 5B and C). We also examined the relative expression of caspase genes. The expression levels of both genes (*caspase-1* and *caspase-3*) were significantly lower in whiteflies infected by PalCuCNV and mPalCuCNV than in whiteflies infected by TYLCV or mTYLCV (Fig. 5D). Similarly, Western blot results showed that accumulation of cleaved-caspase-3 was abundant in whiteflies infected with TYLCV and mTYLCV but barely detected in whiteflies infected with either PalCuCNV or mPalCuCNV (Fig. 5E). These data suggest that the viral CP sequence is not the determinant for the induction of apoptosis.

### Autophagy and apoptosis are independently induced during viral infection.

**FIG 6:**
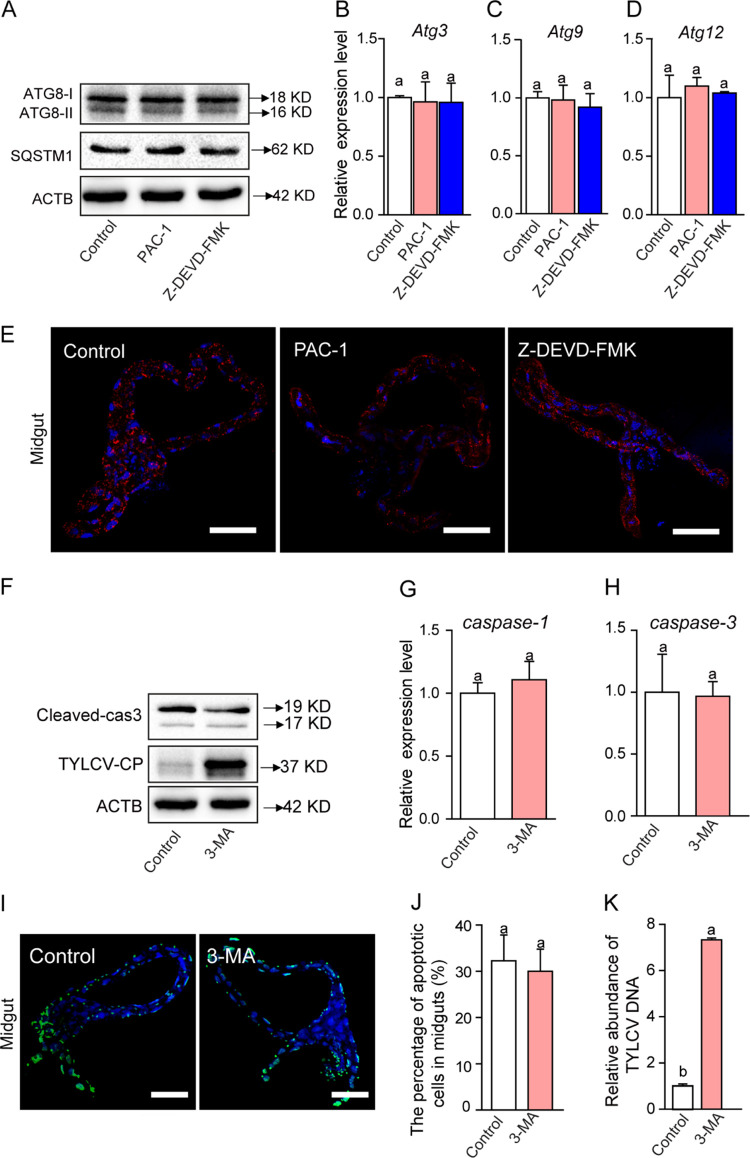
TYLCV infection independently induces autophagy and apoptosis. (A to D) After feeding on TYLCV-infected tomato plants for 24 h, whiteflies were treated with apoptosis inhibitor Z-DEVD-FMK, with apoptosis activator PAC-1, or with DMSO control for 12 h and then transferred to feed on cotton plants for 120 h before being collected for immunoblot analysis. (A) Immunoblot analysis of ATG8 and SQSTM1 in whiteflies. (B to D) Relative expression levels of *Atg3* (B), *Atg9* (C), and *Atg12* (D) were measured by qRT-PCR with *ACTB* expression as the internal control. (E to H) Effects of autophagy inhibitor on the apoptosis pathway. Whiteflies were fed on virus-infected tomato plants for 24 h, treated or not treated with 3-MA for 24 h, and then transferred to feed on cotton plants for 120 h before being collected for analysis. (E) Accumulation of autophagosomes in midguts of whiteflies as detected by immunofluorescence microscopy. Red indicates DyLight 549 staining of autophagosomes. For each of the three treatments, 10 midguts were dissected, and representative images are shown. Bar, 100 μm. (F) Immunoblot analysis of cleaved-caspase-3 and CP in whiteflies treated or not treated with 3-MA. ACTB is the loading control. (G and H) Relative expression levels of *caspase-1* (G) and *caspase-3* (H) were measured by qRT-PCR with *ACTB* and *EF-1a* expression as the internal control. (I, J, and K) Effects of autophagy on apoptosis. (I) Images of guts of whiteflies that were treated with autophagy inhibitor 3-MA, dissected, fixed, and labeled with TUNEL to show apoptosis. For 3-MA treatment and control, 10 midguts were dissected and analyzed, respectively, and representative images are shown. Bar, 100 μm. (J) Proportions of apoptotic cells (number of TUNEL-positive cells/number of DAPI-positive cells) in midguts. (K) TYLCV genomic DNA measured by qPCR. In panels B, C, D, G, H, J, and K, data are mean ± SE. Different letters above the columns in panels B, C, and D (ANOVA, followed by Bonferroni test) or above the two columns in panels G, H, J, and K (Student’s two-tailed *t* test) indicate significant differences.

Two main programmed cell death pathways, autophagy and apoptosis, generally switch from one to the other in a mutually exclusive manner (35). Previously, we found that autophagy in whiteflies induced by TYLCV infection had a negative impact on viral accumulation (12). Therefore, we hypothesized that virus accumulation in whitefly via activation of apoptosis might repress TYLCV-induced autophagy. To investigate the cross talk between apoptosis and autophagy in this system, we fed whiteflies with apoptosis inhibitor (Z-DEVD-FMK) or inducer (PAC-1). Then, we examined TYLCV-induced autophagy by Western blot analysis. The level of ATG8-II (an autophagy-specific protein marker) and SQSTM1 remained unchanged in the inhibitor-fed, the inducer-fed, and the control whiteflies (Fig. 6A). Meanwhile, the expression levels of the autophagy-related genes (*Atg3*, *Atg9*, and *Atg12*) showed no significant changes between the treated and control whiteflies (Fig. 6B, C, and D). We also examined the accumulation of autophagosomes in whitefly guts using confocal immunofluorescence. After Z-DEVD-FMK or PAC-1 treatment, the numbers of autophagosomes showed no significant change (Fig. 6E). These results indicated that activation of apoptosis had no effect on the induction of autophagy.

Next, we treated whiteflies with an autophagy inhibitor (3-MA) (12) and examined its effect on apoptosis. Western blot results showed that the expression level of cleaved-caspase-3 protein remained unchanged between 3-MA-fed and control whiteflies (Fig. 6F). The expression of *caspase-1* and *caspase-3* did not change significantly (Fig. 6G and H). Similarly, apoptotic cells detected by TUNEL staining in the midguts of 3-MA-treated whiteflies exhibited no significant change (Fig. 6I and J), suggesting that treatment with autophagy inhibitor had no effect on the induction of apoptosis. Interestingly, the quantities of virus genomic DNA (Fig. 6K) and CP (Fig. 6F) increased significantly, a result consistent with that of our previous study (12). These results indicated that TYLCV infection independently activated apoptosis and autophagy pathways.

## DISCUSSION

Apoptosis is well known for its roles in development, maintenance of homeostasis, and response to environmental stresses and pathogen infections. Studies on animal viruses have shown that infection of viruses affects host apoptosis in ways that regulate viral replication and spread (22–24). Despite the general recognition of the significance of apoptosis in host-pathogen interactions, little is yet known regarding whether and how plant viruses modulate apoptosis of their insect vectors for their own survival, replication, and transmission. In this study, we show that infection of insect vector by a plant DNA virus significantly activates the apoptosis pathway in the vector and that the activation of apoptosis promotes viral accumulation and transmission. We further reveal that the apoptosis activated by the plant virus is likely independent of autophagy, the other pathway activated simultaneously by the viral infection, and suppresses viral infection of the insect vector. In addition, we show that viral replication, rather than the viral coat protein, might be a factor in apoptosis activation.

The whole process of horizontal transmission of a begomovirus from one plant to the next by sap-sucking insects takes place when the virus in an infected plant gets access to the insect stylet as the vector insect is feeding, and then moves sequentially from the esophagus to the epithelial cells of the alimentary canal, hemolymph, and the salivary glands and finally is secreted together with saliva when the insect lands and feeds on an uninfected plant (5). As shown in Fig. 2D to F, the quantities of TYLCV in whitefly, in particular the two vital organs midgut and salivary glands of the insect, increased significantly when apoptosis was induced by virus infection. A previous study demonstrated that in the whitefly digestive tract, TYLCV localized mostly to the filter chamber and the descending portion of the midgut (36). Our results showed that apoptosis was significantly more induced in those organs where the virions were more abundant (Fig. 1H). The tissue-specific induction of apoptosis may suggest that in the whitefly, apoptosis is a local cell-autonomous response within individual tissues/cells induced by the presence of the virus and may promote virus accumulation and transmission under certain conditions. We speculate that transmission of tissue-specific signals and time-dependent viral movement/spread in the insect vector may be important in the induction of apoptosis. Further research on queries along this line, such as (i) how induction of apoptosis is associated with the time course of viral infection and (ii) what are the crucial vector immunity factors for triggering apoptosis, will improve understanding of the modulation of apoptosis in insect vectors by plant viruses.

Host plant may be a key factor affecting the vector’s capacity and efficiency in plant virus transmission (37). In our experiments, however, no cleaved-caspase-3 could be detected in whiteflies that were fed on uninfected tomato plants for 24 h and then transferred to feed on cotton, suggesting that virus infection but not the host-plant transfer was critical for apoptosis induction in this virus-vector-plant combination. This observation is in line with that of our previous studies on the same virus-vector-plant combination, where the transfer of the feeding whiteflies from tomato to cotton plants was not found to activate stress-related pathways in the whiteflies (12). Therefore, we infer that apoptosis in the whitefly vector was induced directly by infection of the virus.

Insofar as we are aware, our study is the first attempt to examine the role of apoptosis activated by a plant DNA virus in virus transmission by its insect vector. As apoptosis is known for its general role in defense against viral infection, investigation of apoptosis activated by plant DNA viruses in their insect vectors may produce new insight into the interactions between the two types of organisms. Considering that about one-third of the known plant viruses are DNA viruses, interest on this topic is long overdue. Similarly, studies on apoptosis activated by plant RNA viruses in their insect vectors only started recently. In 2015, Huang et al. (38) reported that apoptosis induced by a plant RNA virus (rice ragged stunt virus) promotes virus transmission by its insect vector, a planthopper. More recently, Chen et al. (39) conducted more detailed investigations on the apoptosis activated by another plant RNA virus (rice gall dwarf virus) and provided substantial evidence for the roles of apoptosis in promoting viral infection in its insect vector, a leafhopper. However, as a whole, not much is yet known about the relationship between plant viruses and apoptosis of their insect vectors, partially due to the scarcity of efficient genetic and molecular methods that are applicable to research on a range of plant viruses. In addition, the diversity of plant viruses makes it challenging to identify and study the effects of products associated with viral genes on insect vector apoptosis. Therefore, studies on apoptosis activated by both plant RNA and DNA viruses will be conducive to facilitating understanding of the interactions among circulative plant viruses, insect vectors, and host plants in general (1, 5, 13, 40).

Many viruses have been shown to hijack host cell responses, including autophagy and apoptosis, for their own benefit (41, 42). Moreover, diverse links exist among these responses, and the cross talk between them could significantly affect the fate of viral particles and the infection. Taking the influenza virus as an example, the M2 integral membrane protein affects host cell apoptosis by blocking autophagosome maturation (43). A previous investigation on the Chikungunya virus provides another case study in which virus-induced autophagy inhibits caspase-dependent cell death (35). In a previous study, we showed that TYLCV infection induced autophagy in whiteflies, which exerts a negative impact on viral accumulation (12). Interestingly, in the experiments conducted in this study, inhibiting autophagy did not affect virus-induced apoptosis and *vice versa*. Besides, virtually no apoptosis or autophagy phenomena could be detected in the nonviruliferous whiteflies (12). Much is yet to be learned about the process and the underlying molecular mechanisms of TYLCV infection in the whitefly. In our experiments, TYLCV appeared to initiate the activation of the autophagy pathway sooner (6 h following entry of TYLCV) than the induction of apoptosis (24 h following TYLCV entry). This temporal separation of autophagy and apoptosis may avoid cross talk between the two mechanisms. However, the results of the experiments using autophagy or apoptosis chemical inhibitors suggest that TYLCV induces autophagy and apoptosis pathways independently. Further work is required to examine whether cross talk between autophagy and apoptosis actually occurs.

As obligatory intracellular parasites, plant viruses overcome the first barrier for colonization in their insect vector by modulating the vectors’ immune responses. Our findings provide evidence that TYLCV, a plant DNA virus, triggers apoptosis in the guts of its insect vector, which promotes virus accumulation in the vector and facilitates virus transmission. The CP of the virus is not the key factor that triggers apoptosis; however, whether other nonstructural proteins of TYLCV produced during virus replication play a role in activating apoptosis remains to be determined. In the virus-insect-plant system examined in this study, the positive effects of apoptosis on TYLCV are independent of the autophagy pathway that was also activated by infection of the virus. Future effort should be made to identify the novel factors of plant viruses that trigger apoptosis in their insect vector.

## MATERIALS AND METHODS

### Whitefly rearing.

The cryptic species MEAM1 (mt*COI* [GenBank accession no. GQ332577]) of the Bemisia tabaci whitefly complex was reared on cotton plants (Gossypium hirsutum L. cv. Zhemian 1793) in insect-proof cages at 26 ± 1°C, 16 h of light, and 8 h of darkness. Cotton plants were sown into pots and were cultivated to the 7- to 8-true-leaf stage for experiments unless otherwise indicated.

### Source and maintenance of plants and viruses.

Clones of TYLCV isolate SH2 (GenBank accession no. AM282874.1), TYLCCNV isolate Y10 (GenBank accession no. AJ319675.1), and PalCuCNV isolate HeNZM1 (GenBank accession no. FN256260.1) were agroinoculated into tomato (Solanum lycopersicum L. cv. Hezuo903) at the 3- to 4-true-leaf stage as in our previous study (12). All cultures were grown in cages held in a greenhouse at 25 to 27°C, 60% relative humidity, and natural lighting supplemented with artificial light for 14 h during the day from 06:00 to 20:00.

### Nucleic acid extraction and quantitative PCR.

Total DNA was extracted from plants and whiteflies using the AquaPure genomic DNA isolation kit (Bio-Rad, Hercules, CA, USA). Total RNA was extracted from the whiteflies using the SV total RNA isolation system ((Promega, Madison, WI, USA). RNA was reverse transcribed to cDNA using a cDNA synthesis kit (TaKaRa, Tokyo, Japan). qPCR was performed on the CFX96 Real-Time PCR Detection System (Bio-Rad, Hercules, CA, USA) with SYBR green detection (TaKaRa, Tokyo, Japan). Primers used in this work are listed in Table S1 in the supplemental material. The experiment was run with three biological replicates, and each gene was run in triplicate.

10.1128/mSystems.00433-20.5 TABLE S1 qRT-PCR primers, related to Materials and Methods. Download Table S1, DOCX file, 0.01 MB. Copyright © 2020 Wang et al. 2020 Wang et al. This content is distributed under the terms of the Creative Commons Attribution 4.0 International license.

### Western blot.

Samples were prepared using radioimmunoprecipitation assay (RIPA) buffer with protease inhibitors and analyzed by Western blotting as previously described (12). Antibodies against TYLCV coat protein (3E_10_) and TYLCCNV coat protein (8D_10_) were provided by the Institute of Biotechnology, Zhejiang University. Commercial antibodies to cleaved-caspase-3, ATG8, SQSTM1, cytochrome *c*, and ACTB (beta-actin) were purchased from Cell Signaling Technology (Cell Signaling Technology, Danvers, MA, USA).

### Terminal uridine nick-end labeling (TUNEL) assay.

TUNEL staining was performed using the *in situ* cell death detection kit (Roche, Basel, Switzerland). For apoptotic cell death analyses, 10 whitefly midguts and 10 salivary glands were dissected in TBS (10 mM Tris-HCl, 150 mM sodium chloride, pH 7.5) and fixed in 4% paraformaldehyde for 2 h at room temperature. The tissues were blocked with 5% bovine serum albumin (BSA) in TBS with 0.1% Tween 20 (TBST) and then incubated with the TUNEL reagents (TdT enzyme/dUTP ratio = 1:10) for 6 h. After washing three times in TBS, the tissues were mounted in Fluoroshield mounting medium with 4′,6-diamidino-2-phenylindole (DAPI) (Abcam, Cambridge, MA, USA) and analyzed under a Zeiss LSM710 confocal microscope (Zeiss, Germany). The number of apoptotic cells (TUNEL signals) was counted, and experiments were performed in triplicate.

### TEM.

For transmission electron microscopy (TEM) analyses, 200 midguts obtained from whiteflies were immediately fixed with 2.5% glutaraldehyde in phosphate buffer (0.1 M, pH 7.0) for 4 h and then fixed with 1% OsO_4_ in phosphate buffer for 2 h. After dehydration using increasing ethanol levels, midguts were transferred to acetone for 20 min and then embedded in LX-112, and sections were stained with uranyl acetate and alkaline lead citrate. Images were observed under a Hitachi H-7650 TEM.

### Confocal immunofluorescence microscopy.

Whitefly guts were dissected in TBS (10 mM Tris-HCl, 150 mM NaCl, pH 7.5) and fixed in 4% paraformaldehyde for 2 h at room temperature. Ten midguts were washed three times in TBS, blocked in 5% milk in TBST (TBS buffer with 0.05% Tween 20), and subsequently incubated with primary antibody. The guts were then washed twice in TBST and incubated with secondary antibody diluted in TBST for 2 h at room temperature. After washing three times in TBST, the samples were mounted in Fluoroshield mounting medium with DAPI (Abcam, Cambridge, MA, USA) and imaged using a Zeiss LSM710 confocal microscope (Zeiss, Germany). All treatments were replicated three times.

### Treatment with apoptosis inhibitor and inducer.

Apoptosis inhibitor Z-DEVD-FMK (Selleck Chemicals, Houston, TX, USA) and inducer PAC-1 (Selleck Chemicals, Houston, TX, USA) were used to investigate the effect of apoptosis on TYLCV infection. The inhibitor and the inducer were dissolved in dimethyl sulfoxide (DMSO). First, 600 adult whiteflies at 3 to 5 days postemergence were fed on virus-infected tomato plants for 24 h to acquire the virus and divided into three groups to be fed with a diet of 10 μM Z-DEVD-FMK with 15% sucrose, 10 μM PAC-1 with 15% sucrose, and 15% sucrose containing 0.1% DMSO, respectively, in cylindrical containers for 12 h. Next, the three groups of whiteflies were transferred to feed on cotton plants for 120 h and then collected for Western blot analysis, immunofluorescence, or qPCR. All treatments were replicated 3 times.

### *caspase-3* gene silencing by RNA interference.

The dsRNA was synthesized using the AmpliScribe T7-Flash transcription kit (Epicentre, Madison, WI, USA) with specific primers. For RNA interference, the sucrose with 200 ng/μl ds*caspase-3* dsRNA was used as the treatment group and the sucrose diet containing 200 ng/μl ds*gfp* was used as a negative control. First, 400 adult whiteflies at 3 to 5 days postemergence were fed on virus-infected tomato plants for 24 h and then were fed with ds*caspase-3* or ds*gfp* in cylindrical containers for 48 h, respectively. Subsequently, the two groups of whiteflies were transferred to feed on cotton plants for 120 h before being collected for Western blot analysis, immunofluorescence, and qPCR assays. All treatments were replicated 3 times.

### Monitoring of apoptosis activation in response to mutant virus infection.

First, 400 newly emerged (3 to 5 days old) whiteflies were fed on the mutant TYLCV or mutant PalCuCNV-infected tomato for 24 h and then transferred onto cotton plants for 120 h. Next, those viruliferous whiteflies were collected for Western blot analysis and immunofluorescence. All treatments were replicated 3 times. The two CP mutant viruses were prepared as described in a previous study (34), and a 141-amino-acid (aa) fragment (aa 82 to 222) of the TYLCV CP region was exchanged with a 140-aa fragment (aa 82 to 221) of the PaLCuCNV CP region.

### Transmission of TYLCV after treatment with apoptosis inhibitor and inducer.

First, 600 adult whiteflies at 3 to 5 days postemergence were fed on the virus-infected tomato for 24 h to acquire the virus, and then the viruliferous whiteflies were collected into three groups of 200 each and fed in a rearing tube with a diet of 15% sucrose containing 10 μM Z-DEVD-FMK, 10 μM PAC-1, or DMSO for 12 h for the two treatments and control, respectively.

To examine virus retention by whiteflies in each of the three treatments (2 treatments plus a control), female whiteflies of each of the three groups, as treated above, were immediately transferred to feed on a cotton plant. On 0, 5, and 10 days after transfer onto cotton, 25 whiteflies were collected from each of the three treatments, and their TYLCV genomic DNA was assessed individually using PCR.

To examine TYLCV transmission by whiteflies in the three treatments, the three groups of whiteflies as treated above were transferred to feed on cotton plants for 120 h. Female whiteflies were then transferred and enclosed using clip cages to feed on the third leaf of an uninfected tomato plant at the 3-true-leaf stage for TYLCV transmission. For each of the three treatments, 25 viruliferous female whiteflies were inoculated individually to 25 plants. After 48 h, the whiteflies were removed, and the plants were sprayed with imidacloprid (50 mg/liter) to kill the eggs. After a further 25 days, the leaves of each tomato plant were collected and subjected to qPCR assays of virus infection and virus abundance in the plant as normalized with tomato actin.

### Cross talk between autophagy and apoptosis.

Apoptosis inhibitor Z-DEVD-FMK (Selleck Chemicals, Houston, TX, USA) and autophagy inhibitor 3-MA (MilliporeSigma, Burlington, MA, USA) were dissolved in DMSO. First, 800 adult whiteflies at 3 to 5 days postemergence were fed on the virus-infected tomato plants for 24 h and then were divided into four groups to be fed with a diet of 15% sucrose containing 10 μM Z-DEVD-FMK (for 12 h), DMSO (for 12 h), 1 μM 3-MA (for 24 h), and DMSO (for 24 h), respectively. Next, the four groups of whiteflies were transferred to feed on cotton plants for 120 h and then collected for Western blot analysis, immunofluorescence, and qPCR. The whole experiment was replicated 3 times.

### Statistical analysis.

For all data of gene expression, percentages of apoptotic cells, the abundance of virus DNA, percentages of mortality, the proportion of whiteflies or plants with virus DNA, and percentages of virus-infected plants, one-way analysis of variance (ANOVA) was applied for the analysis followed by Bonferroni test when there were ≥3 treatments, and Student’s two-tailed *t* test was applied for the analysis when there were only two treatments. Percentage data were arcsine square-root transformed to be used in the analysis, but the original data of percentages are presented. The percentage data of tomato plants showing typical symptoms of TYLCV infection were analyzed with Mann-Whitney U-tests because each of the three treatments had only one replicate. Differences were judged significant when *P* was <0.05.
