# Supplementary figures and images for: Apoptosis in a Whitefly Vector Activated by a Begomovirus Enhances Viral Transmission
Source: mSystems. 2020 Sep 22;5(5):e00433-20. doi: 10.1128/mSystems.00433-20 (PMC7511215; doi:10.1128/mSystems.00433-20)

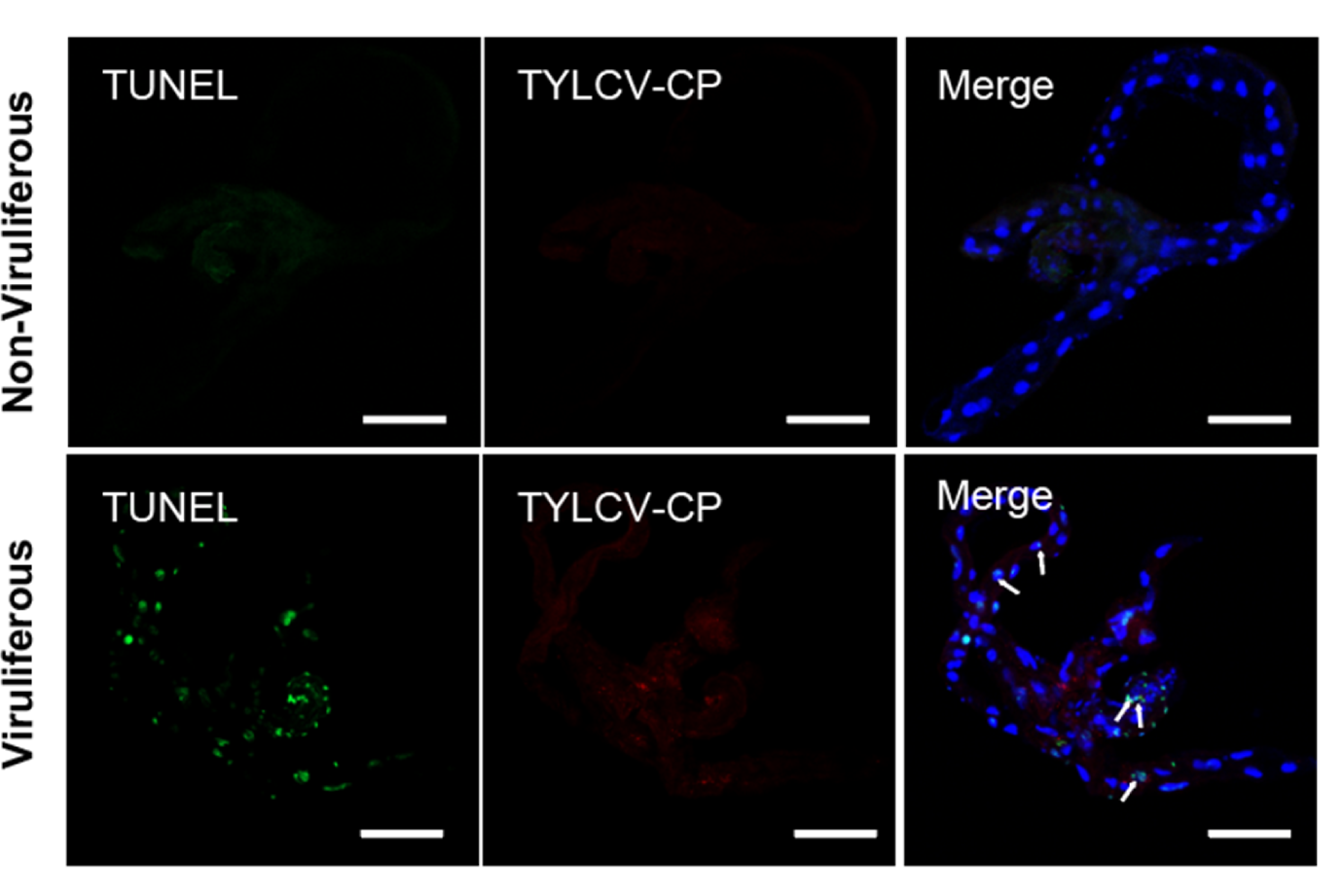

Supplement: FIG S1 [file mSystems.00433-20-sf001.tif]

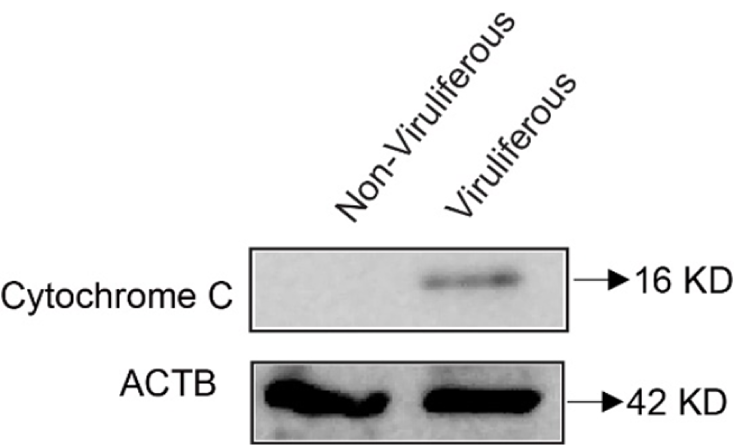

Supplement: FIG S2 [file mSystems.00433-20-sf002.tif]

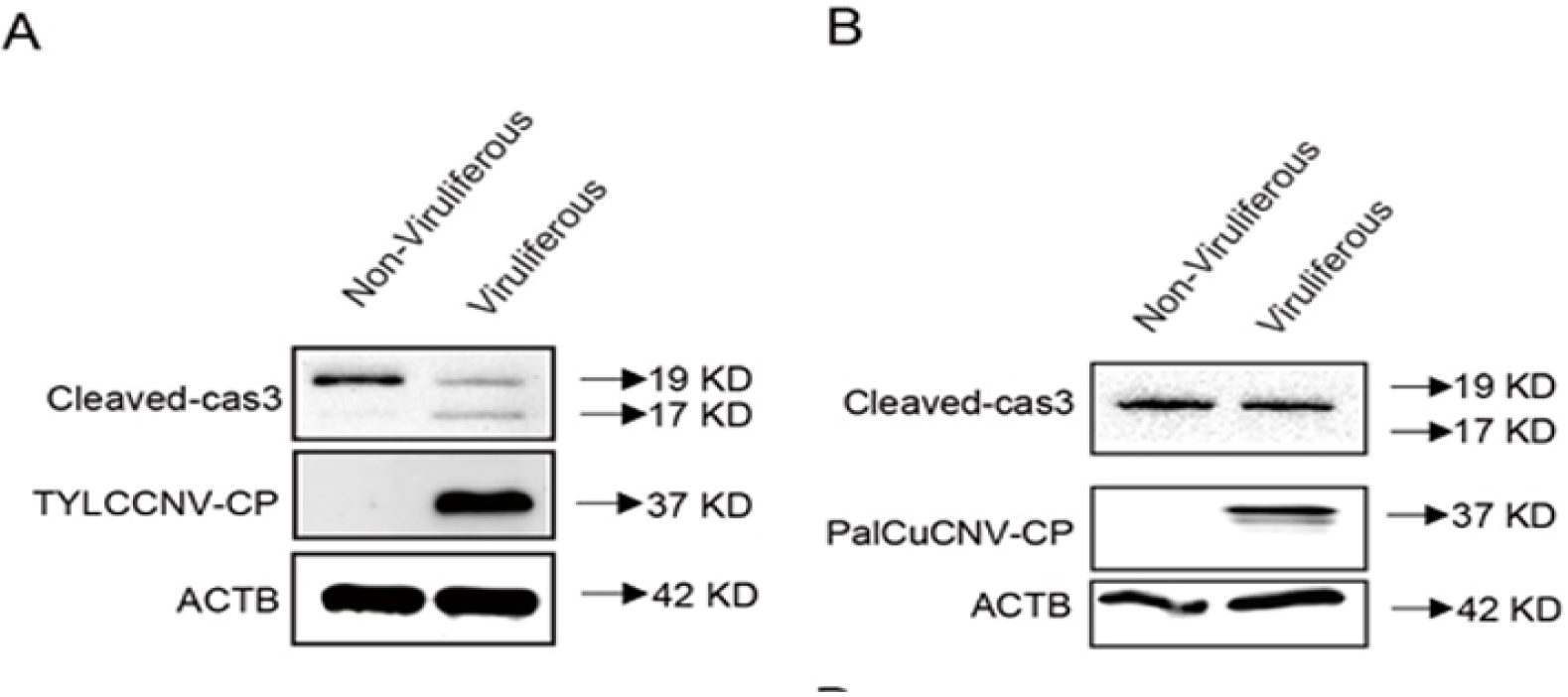

Supplement: FIG S3 [file mSystems.00433-20-sf003.tif]

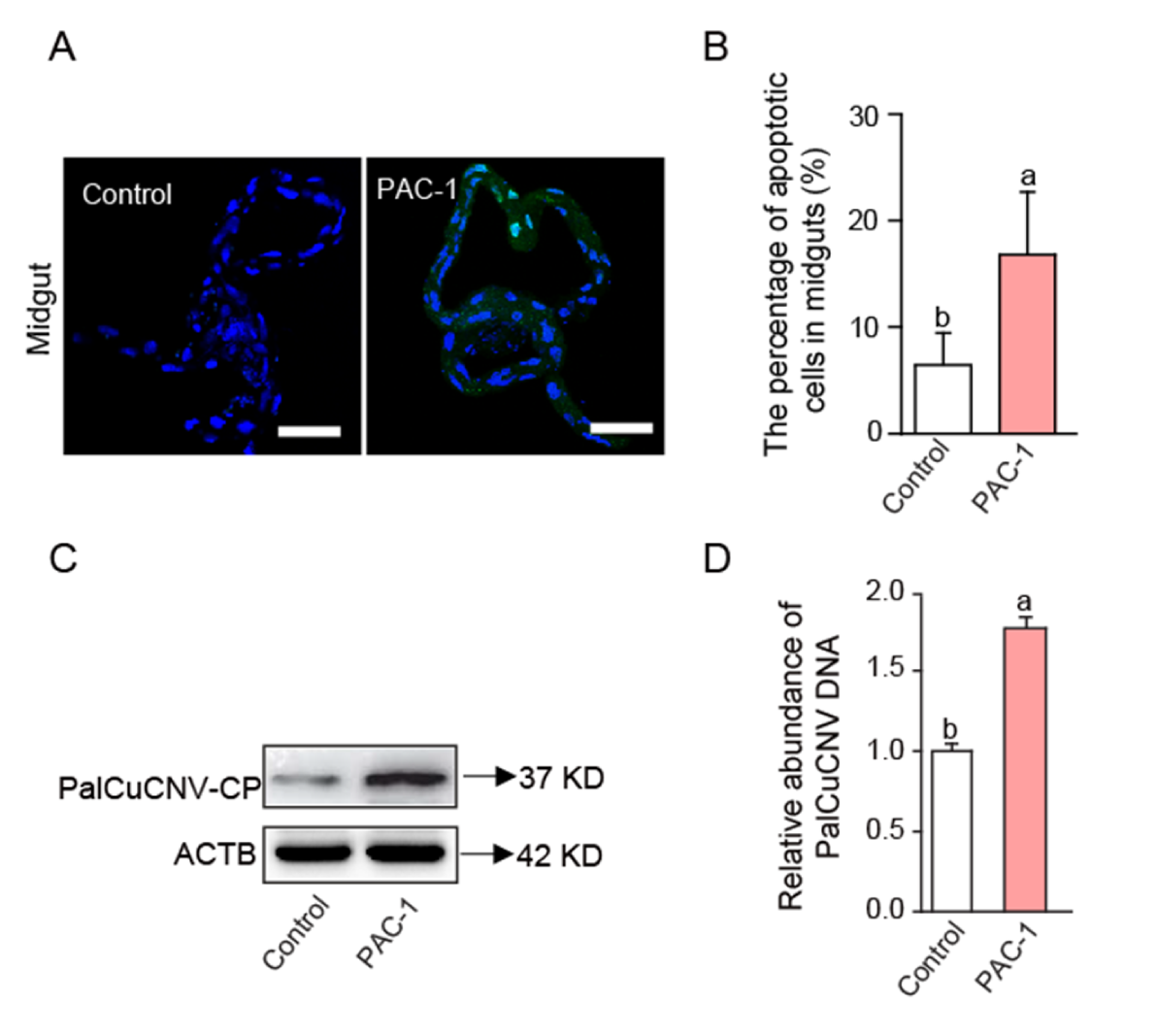

Supplement: FIG S4 [file mSystems.00433-20-sf004.tif]
