# Supplementary material for: Apoptosis in a Whitefly Vector Activated by a Begomovirus Enhances Viral Transmission
Source: mSystems. 2020 Sep 22;5(5):e00433-20. doi: 10.1128/mSystems.00433-20 (PMC7511215; doi:10.1128/mSystems.00433-20)
Supplement: TABLE S1 [file mSystems.00433-20-st001.docx]

| Gene name | Forward primer (5’–3’) | Reverse primer (5’–3’) |
| --- | --- | --- |
| *TYLCV-DNA*  *TYLCV-V1*  *TYLCV-C3* | TCTGTTCACGGATTTCGTTG  GAAGCGACCAGGCGATATAA  TGAGGCTGTAATGTCGTCCA | GCTGTCGAAGTTCAGCCTTC  GGAACATCAGGGCTTCGATA  GCTCCTCAAGCAGAGAATGG |
| *caspase-1* | TGTCTGCCTTTCAAGGGACC | CTTGACTTTGCAACACCGCA |
| *caspase-3* | CATCACGATCAACGGGACCA | TGTCGATGTGCTGCTCGAAT |
| *iap* | TGTGAACTTTTGCCGCGAAG | CCACGATGTGACCACAAGGA |
| *bcl-2* | GGAAAGTCTCGGAGAGTCCTG | GCCACATTCCAATTCCAGTGC |
| *atg3* | CCAGATTGTCTCCAGCAGCA | CGTTTAAGGGAACAGCACTTG |
| *atg9* | TCAGGAAGAAGTACCCCGACA | AAGCACAGATCTGGCCGTAG |
| *atg12* | TCAAAGCCACTGGAAACGC | TCTGGTCTGGAGCAGGAGC |
| *PaLCuCNV-V1* | GGGCAAGATATGGATGGATG | ACCTGACCAAAATCCTGTGG |
| *PaLCuCNV-C3* | AGAAACGCCAAGTCTGAGGA | ATTCAACACCAACCACGACA |
| ds*gfp* | TAATACGACTCACTATAGGGAGACCACTGACCCTGAAGTTCATCTGC | TAATACGACTCACTATAGGGAGACCACGTCTTGTAGTTGCCGTCGTC |
| ds*caspase-3b* | TAATACGACTCACTATAGGGAGAT CATCACGATCAACGGGACCA | TAATACGACTCACTATAGGGAGAT TGTCGATGTGCTGCTCGAAT |
| *β-actin* (whitefly)  *EF-1a* (whitefly)  *β-actin*(tomato) | TCTTCCAGCCATCCTTCTTG  TAGCCTTGTGCCAATTTCCG  TGGAGGATCCATCCTTGCATCAC | CGGTGATTTCCTTCTGCATT  CCTTCAGCATTACCGTCC  TCGCCCTTTGAAATCCACATCTGC |

**Supplementary Table S1. qRT-PCR primers, related to Methods.**
